# The direct-to-indirect band gap crossover in two-dimensional van der Waals Indium Selenide crystals

**DOI:** 10.1038/srep39619

**Published:** 2016-12-23

**Authors:** G. W. Mudd, M. R. Molas, X. Chen, V. Zólyomi, K. Nogajewski, Z. R. Kudrynskyi, Z. D. Kovalyuk, G. Yusa, O. Makarovsky, L. Eaves, M. Potemski, V. I. Fal’ko, A. Patanè

**Affiliations:** 1 School of Physics & Astronomy, The University of Nottingham, Nottingham, NG7 2RD, UK; 2 Laboratoire National des Champs Magnétiques Intenses, CNRS-UGA-UPS-INSA-EMFL, 25 rue des Martyrs, 38042 Grenoble, France; 3 National Graphene Institute, The University of Manchester, Manchester, M13 9PL, UK; 4 Institute for Problems of Materials Science, The National Academy of Sciences of Ukraine, Chernivtsi, 58001, Ukraine; 5 Department of Physics, Tohoku University, Sendai 980-8578, Japan

## Abstract

The electronic band structure of van der Waals (vdW) layered crystals has properties that depend on the composition, thickness and stacking of the component layers. Here we use density functional theory and high field magneto-optics to investigate the metal chalcogenide InSe, a recent addition to the family of vdW layered crystals, which transforms from a direct to an indirect band gap semiconductor as the number of layers is reduced. We investigate this direct-to-indirect bandgap crossover, demonstrate a highly tuneable optical response from the near infrared to the visible spectrum with decreasing layer thickness down to 2 layers, and report quantum dot-like optical emissions distributed over a wide range of energy. Our analysis also indicates that electron and exciton effective masses are weakly dependent on the layer thickness and are significantly smaller than in other vdW crystals. These properties are unprecedented within the large family of vdW crystals and demonstrate the potential of InSe for electronic and photonic technologies.

To date, a wide variety of two-dimensional (2D) van der Waals (vdW) crystals have been investigated, including transition metal dichalcogenides (TMDCs), black phosphorus (bP), and hexagonal boron nitride (hBN), and exploited as single crystals or in combination with graphene to create functional devices^1^,^2^,^3^. Among the vdW crystals, the metal monochalcogenide III-VI compound, InSe, has emerged as a promising 2D semiconductor. The γ-polytype phase of InSe has a direct optical band gap that increases due to quantum confinement of the charged carriers when the number of layers, *L*, in the crystalline sheet is reduced^4^,^5^,^6^. Also, at small *L*, the energy-momentum relation of the valence band (VB) takes the form of an inverted “Mexican hat”. Near the VB edge, the constant energy contours have the form of a ring in *k-*space and the 2D density of states develops a one-dimensional Van Hove singularity^7^,^8^,^9^, a property that could lead to tuneable magnetism, superconductivity, and enhanced thermoelectricity^10^,^11^. These findings have the potential to extend further the prospects of InSe as a material for several technologies and devices, which range from field effect transistors (FETs)^12^,^13^ with record high room temperature electron mobility (*μ* = 0.2 m^2^V^−1^s^−1^)^13^ to bendable^14^ and high-gain^15^ photodetectors, image sensors^16^, and photon sources^17^.

Despite this burgeoning research, there are fundamental and technological aspects of 2D InSe that remain elusive. The observation of photoluminescence (PL) from few-layer InSe (<7 layers) has been demonstrated only recently in *encapsulated (e.g.* InSe/hBN)^13^ and *texturised (e.g.* InSe/SiO_2_)^18^ layers. In the latter case, the surface of InSe flakes is bent by SiO_2_ nanoparticles trapped between the flakes and the substrate, thus enhancing the optical emission due to increased light scattering and modified dipolar selection rules^18^. However, it is not yet known if thin layers of *non-texturized* InSe are optically active when exposed to air. Furthermore, fundamental band parameters, such as electron, hole and exciton masses, which are well known in bulk InSe^19^,^20^, are largely unknown in 2D InSe. More generally, still more work is needed to deepen our understanding of the unique electronic band structure of this 2D crystal, which underpins not only future research, but also applications of InSe and its competitiveness with other vdW crystals, such as TMDCs and bP.

Here we report on the electronic and optical properties of exfoliated InSe nanosheets under light illumination with the electric field polarized in the plane of the layers and in magnetic field, *B*, up to 30 T applied in the Faraday geometry. We demonstrate PL emission from InSe layers exfoliated in air over an extended range of layer thicknesses, down to *L* = 2 layers, a tuneable band gap energy ranging from ~1.3 eV (*L* > 20 layers) to 2 eV (*L* = 2 layers), and quantum dot-like optical emissions distributed over a wide range of energy. We observe no emission in single-layer InSe, which we attribute to a selection rule, which forbids optical transitions for light polarized in the plane of the layers due to the mirror symmetry of the single layer crystal. Our data and density functional theory (DFT) calculations also indicate than electron and exciton in-plane effective masses are weakly dependent on the layer thickness and are significantly smaller than in other vdW crystals. These properties combined with the chemical stability in air of InSe expands significantly the range of potential applications of 2D vdW crystals.

## Results

### Band structure and Landau level quantization

**Figure 1:**
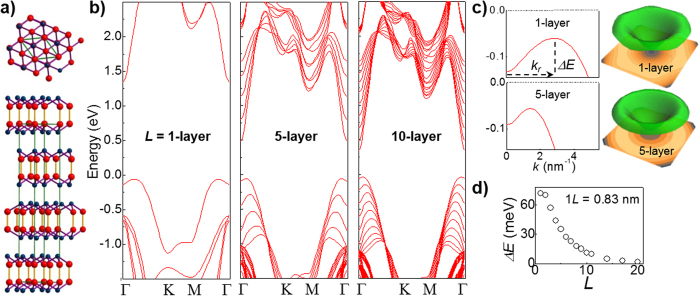
Band structure of two-dimensional InSe. (**a)** Crystal structure of *γ*-InSe in the *ab*-plane (top) and along the *c*-axis (bottom). (**b)** Band structure of InSe with *L* = 1, 5 and 10 layers. (**c)** Valence band (VB) over a narrow range of energy and *k*-values close to the Γ-point of the Brillouin zone for *L* = 1 and 5 layers. The insets show contour plots of the VB energy dispersion in the *k*-plane centred at Γ. For *L* = 1 layer, the valence band comprises of six maxima that are arranged in a circle around Γ; this anisotropy is not observed for *L* = 5 layers. (**d)** Dependence on *L* of the energy splitting, *ΔE*, between the VB maximum and the VB edge at Γ.

Figure 1a shows the crystal structure of the γ-polytype phase of InSe. The primitive unit cell contains three layers each of which has a thickness of *t* = 8.320 Å (*L* = 1 layer) and consists of four covalently bonded monoatomic sheets in the sequence Se-In-In-Se; along the *c*-axis, the primitive unit cell has a lattice constant *c* = 24.961 Å; within each *a-b* plane atoms form hexagons with lattice parameter *a* = 4.002 Å. Figure 1b shows the electronic band structure in the Brillouin zone for InSe crystals containing 1, 5, and 10 layers, as derived from DFT (see Methods). It reveals that when *L* is reduced, the conduction band minimum (CBM) remains at the Γ*-*point and shifts to high energy; in contrast, the valence band maximum (VBM) moves away from Γ towards the K*-*points, and the VB takes the form of an inverted “Mexican hat” (Fig. 1c). With decreasing *L*, the energy interval *ΔE* between the valence band edge at Γ and the VBM increases from 0 to ~70 meV (Fig. 1d); correspondingly, the VBM shifts from Γ to about 30% of the Γ-K wave-vector. Thus a direct-to-indirect band gap crossover occurs gradually with decreasing *L* due to a qualitative change of the VB at *L* < 20 layers.

**Figure 2:**
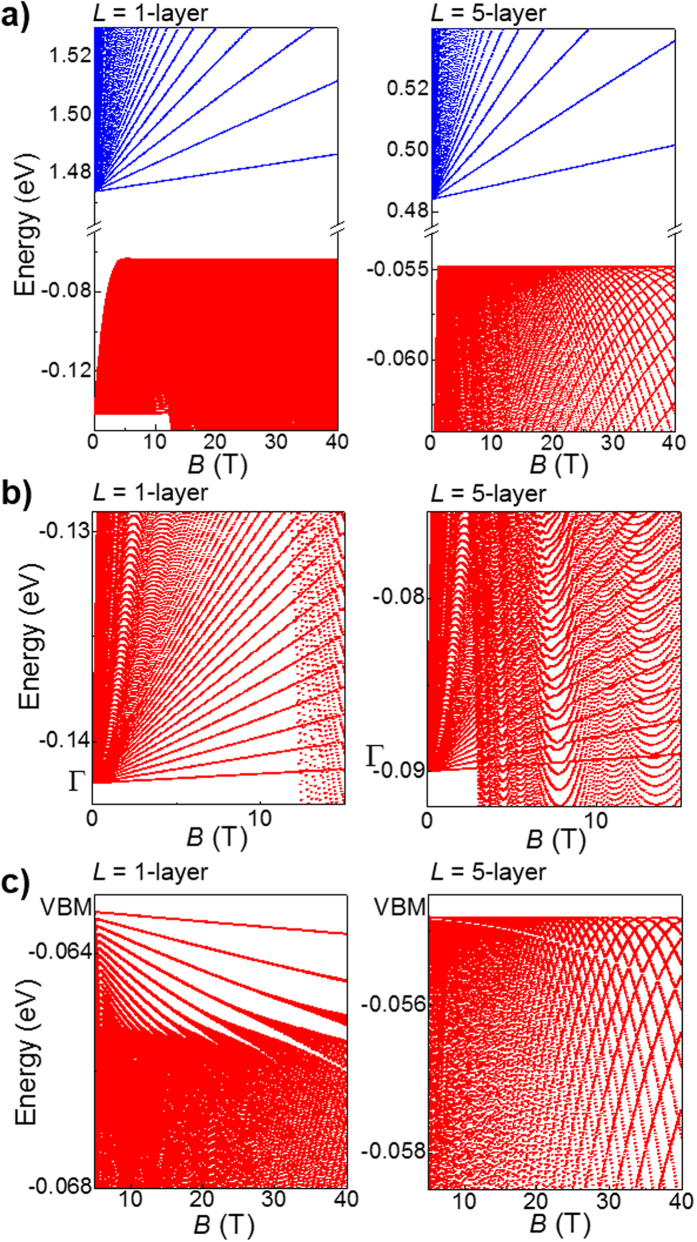
Landau level quantization in two-dimensional InSe. (**a)** Energy of the Landau levels (LLs) versus *B* for electrons (blue) and holes (red) in the conduction and valence bands (*L* = 1 layer and 5 layers). The hole LLs are zoomed-in in parts b-c. (**b,c)** Hole LLs versus *B* at energies around Γ (**b**) and the valence band maximum, VBM (**c**) for *L* = 1 layer (left) and 5 layers (right). In parts (**b**) and (**c**), Γ and VBM indicate the VB edge at Γ and VBM at *B = *0.

To calculate the Landau level (LL) spectra, we fit analytical expressions to the CB and VB energy dispersions, and introduce the vector potential using the Peierls substitution (see Methods). Figure 2a shows the calculated LLs near the edges of the CB and VB for *L* = 1 and 5 layers. For the CB, the LL spectrum consists of a series of well-separated pure Landau harmonics. A similar LL spectrum was calculated recently for *L* = 1 layer^21^. For the VB, the LL spectrum comprises instead of two series of closely-spaced levels that cross and admix (Fig. 2b,c). This dense LL spectrum arises from the magnetic quantization of the weakly dispersed “Mexican hat” VB. The unusual energy dispersion of the VB leads to a cyclotron hole mass, 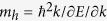, which is negative around the Γ-point, changing to a positive value at the VBM. Table 1 compares our calculated values of the electron (hole) in-plane cyclotron mass at Γ (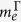 and 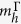) and at the VBM (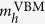) for different *L* with those for bulk InSe from the literature^19^,^20^: 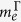 increases with decreasing *L*, but remains close to the in-plane cyclotron mass measured in the bulk (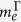
*/m*_*e*_ = 0.138, where *m*_*e*_ is the free electron mass); close to the Γ-point, where the holes have a CB-like energy dispersion (Fig. 2b), the negative hole cyclotron mass 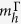 has absolute value that is smaller than the hole mass at the VBM, 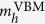, but significantly larger than the electron cyclotron mass at Γ, 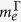.

Near the edge of each band in the CB and VB, the LL energies for electrons and holes can be approximated by the relation 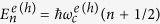, where *n* is an integer, 
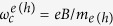
 is the cyclotron frequency and *m*_*e(h*)_ is the electron (hole) in-plane cyclotron mass. Due to the change of sign and magnitude of *m*_*h*_ with increasing *k*-vector away from the Γ-point, the VB LLs overlap and admix, leading to the complex LL spectra shown in Fig. 2b,c. In particular, since the VB energy dispersion and hole mass vary with *L*, the LL admixing tends to shift at higher *B* with decreasing *L*.

### Photoluminescence and magneto-photoluminescence

**Figure 3:**
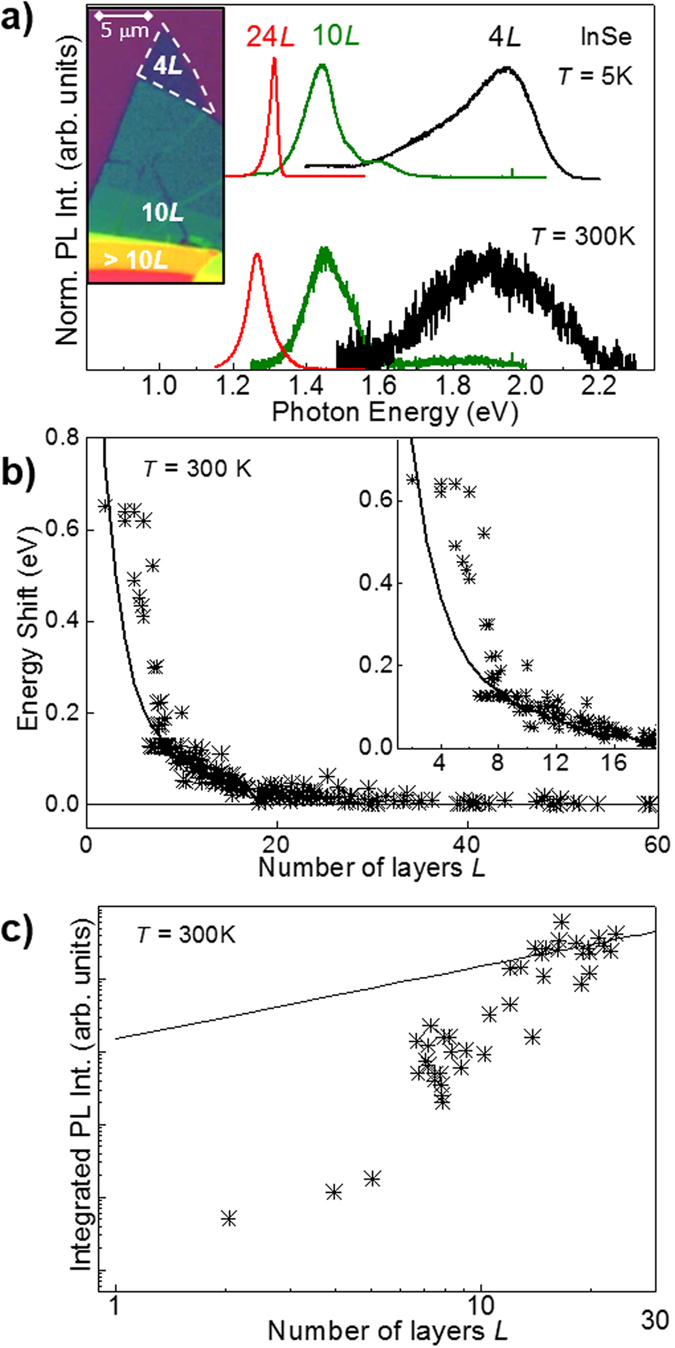
Quantum energy shift of band-to-band optical transitions in InSe. (**a)** Normalized μPL spectra for InSe nanosheets with *L* = 4, 10 and 24 layers at *T* = 5 K and 300 K. The inset is an optical image of the nanosheet with sections of thickness *L* = 4, 10 and >10 layers. (**b)** Calculated (lines) and measured (symbol) energy increase of the band gap of InSe with decreasing *L*. Inset: Same as in part a) but for small *L*. (**c)** PL integrated intensity versus *L*. The line represents the relation *αL*, where *α* is a constant absorption coefficient.

At *T* = 300 K and *B* = 0 T, the PL peak energy increases from 1.25 eV for bulk crystals to ~2 eV for flakes with *L* = 2 layers, in agreement with the DFT calculations (Fig. 3a,b). Correspondingly, the intensity of the PL signal decreases markedly as *L* is decreased below *L *~ 12 layers (Fig. 3c), much more strongly than expected from a reduction in the thickness of the optically absorbing material (see line in Fig. 3c). No PL emission could be detected in the single layer flakes. The measured shift of the PL emission is among the largest reported for vdW crystals and comparable to those demonstrated for encapsulated^13^ and texturized^18^ InSe, and bP^22^.

For thin flakes (*L* < 20 layers), the energy position of the main PL emission changes only slighly when the temperature is decreased from 300 K to 5 K (Fig. 3a). Previous studies have demonstrated that this behaviour arises from the contribution to the low *T* PL of carrier recombination from localized states that become ionized at high temperature^23^. These localized states arise from native donors, acceptors, and donor-acceptor pairs. They have binding energies that depend on the location of the impurities within the layers and increase with decreasing *L* due to the compression of the carrier wavefunction along the *c*-axis.

**Figure 4:**
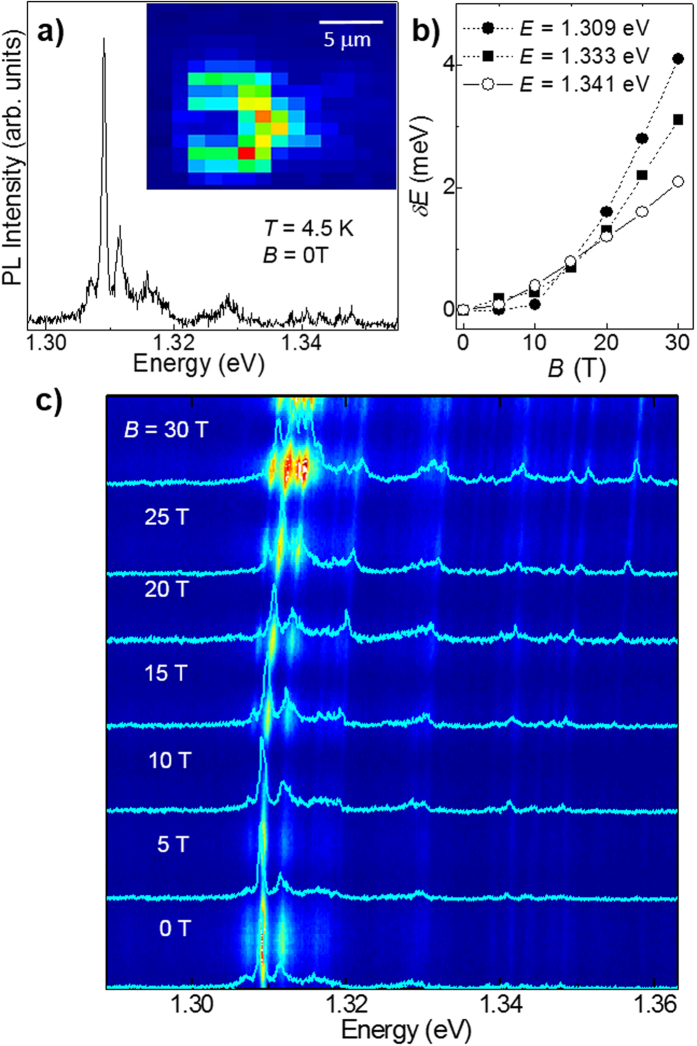
Narrow photoluminescence emission lines in InSe. (**a)** Narrow μPL lines at magnetic field *B* = 0 T and *T* = 4.2 K. The inset shows a μPL map acquired at the energy of the μPL line at 1.309 eV. (**b)** Energy shift *δE* versus *B* for three representative μPL lines. The dotted lines are guides to the eye. (**c)** Colour map of the PL intensity versus *B* and photon energy. The μPL spectra for representative *B* are overlapped on the colour map. The magnetic field is parallel to the *c*-axis of InSe and the experiment is conducted in the Faraday geometry.

The localization of excitons around impurities and/or crystal defects can also lead to narrow (~0.5 meV) quantum dot-like μPL emission lines distributed over a wide range of energy, as shown in Fig. 4a for an InSe flake with *L *~ 20 layers. These narrow lines arise from well-defined hot spots on the flake (see Fig. 4a-inset for the μPL map acquired at the energy of the μPL line at 1.309 eV) and are only weakly affected by *B* up to ~10 T, which corresponds to a magnetic length 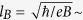 8 nm (Fig. 4b,c). The measured diamagnetic shifts *δE* range from 2 to 4 meV at *B* = 30 T (see Fig. 4b) and are smaller than the shift reported for the free exciton in bulk InSe (*δE* = 5.1 meV at *B* = 30 T)^24^. Furthermore, a spin-splitting of the lines cannot be resolved even at *B* = 30 T. This indicates that the effective *g*-factor and corresponding splitting (*gμ*_B_*B*) are small compared to those (*g* > 9 and *gμ*_B_*B* > 15 meV at *B* = 30 T) measured for localized excitons in TMDCs^25^.

**Figure 5:**
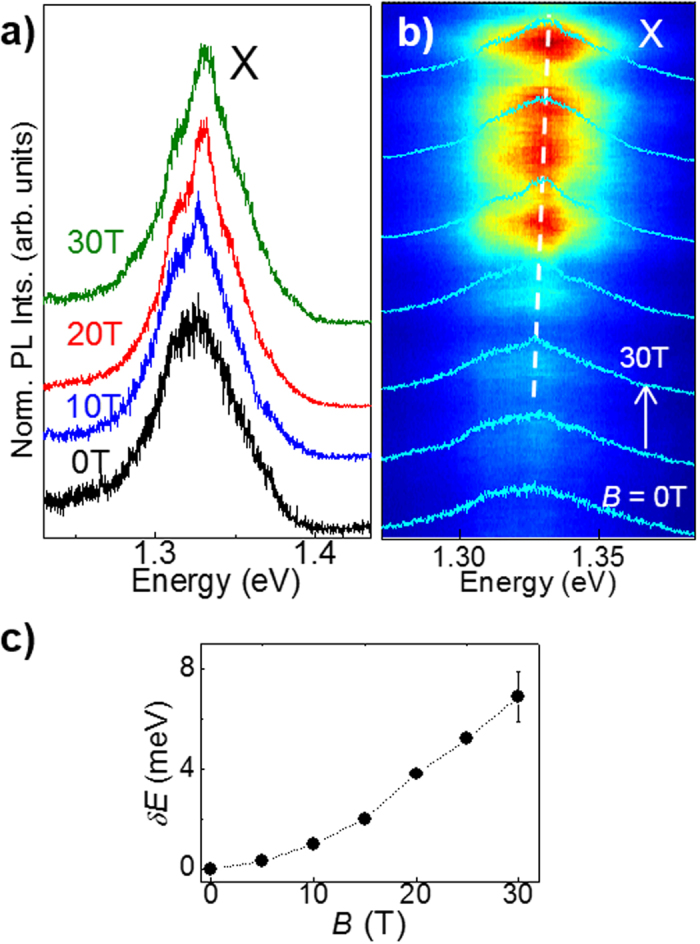
Magneto-photoluminescence of bulk InSe. (**a**) Normalized μPL spectra at various magnetic fields *B* = 0, 10, 20 and 30 T for a bulk InSe flake in the Faraday geometry and *B* parallel to the *c*-axis (*T* = 4.2 K). (**b)** μPL spectra overlapped over a colour map of the PL intensity versus *B* and photon energy. The PL spectra are shown for *B* increasing from 0 to 30 T in steps of 5 T. The dotted line shows the energy shift of the X-band. (**c)** Energy shift *δE* of the X-band versus *B*. The dashed line is a guide to the eye.

We now examine the effect of the magnetic field *B* on the μPL emission of those InSe flakes for which quantum dot-like narrow μPL lines are absent. First, we consider bulk-like InSe flakes (*L* > 20 layers). For the sample shown in Fig. 5a,b, the low *T* (4.2 K) PL band emission, labelled X, is centred at ~1.33 eV, at the energy of the free exciton in bulk InSe^24^. At *B* > 15 T, the intensity of the X-band increases and its shift *δE* has a linear dependence on *B, i.e. δE* = σ_//_*B*, where σ_//_ = 4 × 10^−4^ eV/T (Fig. 5b,c).

**Figure 6:**
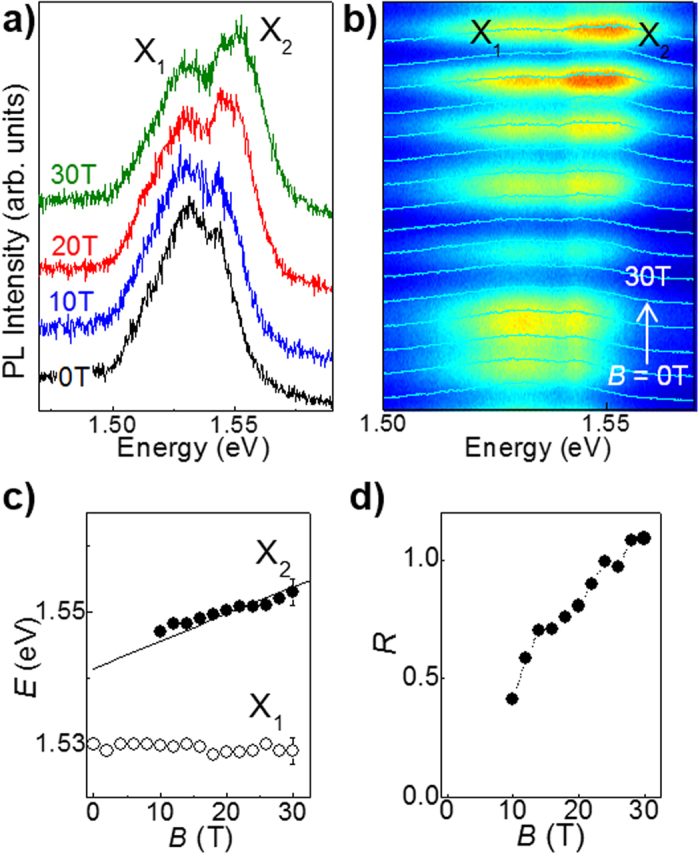
Magneto-photoluminescence of InSe nanosheets. (**a**) Normalized μPL spectra at magnetic field *B* = 0, 10, 20, and 30 T for an InSe flake with *L *~ 5 layers in the Faraday geometry and *B* parallel to the *c*-axis (*T* = 4.2 K). (**b**) μPL spectra overlapped over a colour map of the PL intensity versus *B* and photon energy. The oscillations in the μPL intensity with *B* are due to the use of optical fibres (see Methods). The spectra are shown for *B* increasing from 0 to 30 T in steps of 2 T. (**c**) Energy peak position of the X_2_ and X_1_ bands and (**d)** ratio of their intensity, *R*, versus *B*. The line in part c) describes the linear shift of the X_2_ band, *i.e. E *∝ 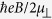, where *μ*_⊥_ = 0.14*m*_*e*_ is the reduced mass of the exciton. The dotted line in part d) is a guide to the eye.

The low *T* (4.2 K) μPL spectra for an InSe flake with *L *~ 5 layers and *B* up to 30 T are shown in Fig. 6a,b. At *B* = 0 T the main μPL band, X_1_, is centred at 1.53 eV, significantly higher than for our bulk flakes with *L* > 20 layers. With increasing *B*, a stronger PL emission band, X_2_, emerges at higher energy. Figures 6c,d show the *B*-dependence of the energy positions of the X_2_ and X_1_ PL peaks and the corresponding ratio of intensities *R* derived from fits to the PL spectra by two Gaussian lineshapes. The relative weight of the X_1_ and X_2_ bands changes with increasing *B*, with the intensity shifting significantly to the higher energy peak X_2_. Also, whereas the X_1_ band hardly shift in energy, consistent with carrier recombination from localized states, the X_2_ band shows an approximately linear blue-shift with increasing *B, i.e. δE* = σ_//_*B*, where σ_//_ = 4 × 10^−4^ eV/T at *B* > 15 T. This dependence is very similar to that measured for the free exciton in our bulk InSe flakes.

## Discussion

At *B* = 0 T and *T* = 300 K, the PL emission of exfoliated InSe layers undergoes a strong blue-shift to higher photon energies with decreasing *L* (Fig. 3b). Although our DFT underestimates the band gap energy, *E*_*g*_, the calculated increase of *E*_*g*_ with decreasing *L* (line in Fig. 3b) is in good agreement with the measured values. The larger measured energy shift for thin InSe layers suggests either that DFT may not be accurate at small *L* (<8 layers) and/or that carriers are confined within an effective thickness that is smaller than that measured by atomic force microscopy. The latter possibility may be due to a thin surface film into which the carrier wave function does not penetrate. Such a film can originate from the contamination of the InSe surface due to the chemisorption of water and oxygen molecules on a low density of surface dangling bonds^26^. Furthermore, a corrugation of the surface can cause an additional in-plane quantum confinement. This can influence the energy position and linewidth of the PL emission, and also induce quantum dot-like emissions: the band gap energy changes by ~0.1 eV with varying *L* by a single layer at *L* = 4 layers (Fig. 3b-inset).

The DFT calculations of the band structure of 2D InSe indicate the emergence of a weakly dispersed “Mexican hat” VB for *L* < 20 layers, accompanied by a direct-to-indirect band gap crossover: the separation *ΔE* between the VBM and the VB-edge at Γ increases from 0 to 70 meV with decreasing *L* (Fig. 1d). As shown in Fig. 3c, the PL signal is observed for a range of layer thicknesses and is not suppressed at *L* = 20 layers, although it weakens steadily with decreasing *L*. To explain this finding, we note that optical transitions involve hole states with a range of *k*-vectors and energies near the VBM. For example, for *L* = 10 layers, the thermal energy of holes at *T* = 300 K (*k*_*B*_*T = *26 meV) is larger than *ΔE* = 11 meV. Thus InSe remains effectively a “quasi-direct” semiconductor for a range of thicknesses *L* such that *k*_*B*_*T* > *ΔE, i.e.* for *L* > 6 layers at *T* = 300 K. Although this argument accounts qualitatively for the data, one should also consider additional effects. The atomic orbitals change with decreasing *L*, leading to a corresponding decrease of absorption coefficient^18^. In particular, for the case of single layers, the optical signal is suppressed for light polarized in the layer plane due to crystal symmetry^7^. The mirror-plane (*z* ⇒−*z*) symmetry of the single layer implies that the valence/conduction band edge states have even/odd wave functions; this makes the lowest energy electron-hole excitation optically inactive for in-plane polarized light.

Whereas the room temperature PL emission is dominated by band-to-band transitions, the low temperature PL spectra reveal carrier localization and quantum-dot like emissions, anagolous to those reported in atomically thin TMDC layers^25^,^27^,^28^,^29^. Here we use high magnetic fields to discrimintate between contributions to the PL emission due to localized and delocalized excitons. As shown in Fig. 6, the intensity and energy peak position of the low energy band X_1_ in the 5-layers InSe is weakly affected by *B*, suggesting a strong spatial localization of the photoexcited carriers (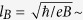
5 nm at *B* = 30 T). On the other hand, the enhancement of the higher-energy band X_2_ with increasing *B* and its energy shift indicate a contribution from excitons that are less strongly confined. At *B* > 15 T, we describe the linear energy shift of the X_2_ band (Fig. 6c) in terms of an interband transition between electron and hole LLs with *n* = 0, *i.e. E* ∝ 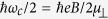, where *μ*_⊥_ = (0.14 ± 0.01)*m*_*e*_ is the in-plane reduced mass of the exciton. Since holes are much heavier than electrons (Table 1), the shift of the exciton line is determined by the LL quantization of the lighter electrons and is approximatively linear. The nearly flat-band dispersion of the holes suggests that holes are easily localised by any defects in the 2D crystal, hence, their diamagnetic energy shift is negligibly small. The value of *μ*_⊥_ = (0.14 ± 0.01) *m*_*e*_ is close to the electron cyclotron mass calculated by DFT for *L* = 5 layers (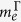 = 0.13*m*_*e*_) and measured by magneto-transport (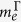 = 0.14*m*_*e*_)^13^. These values also coincide with those measured in our bulk flakes with *L* > 20 layers and those reported in the literature for bulk InSe (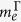 = 0.138*m*_*e*_^20^ and *μ*_⊥_ = 0.14*m*_*e*_^23^). Thus both theory and experiment indicate that for layer thicknesses down to ~5 layers, the exciton and electron in-plane masses are weakly dependent on *L*. In particular, due to the heavy hole mass, the quantization of the electron motion in the layer plane rules out the disorder-induced localization of the excitons observed in these flakes.

In summary, we have demonstrated that InSe nanoflakes in air have an optical response that can be tuned from the NIR to the visible spectrum with decreasing flake thickness down to 2 layers. In contrast to TMDCs that have a direct band-gap only in films with one or two layers, InSe has a direct-band gap over a wide range of layer thicknesses. Furthermore, even when the crystal becomes an indirect band gap semiconductor, it remains optically active due to its weakly dispersed valence band. These properties relax the stringent condition required for the optical activity of single- or bi-layer TMDCs and can facilitate the use of InSe for the fabrication of sensitive photodetectors and efficient light emitters over a broad spectral range. Our magnetic field studies and DFT calculations of the band structure reveal relatively small exciton and electron masses in the layer plane, close to those for bulk InSe and smaller than for TMDCs^30^. The small electron mass and its weak dependence on the layer thickness is fully consistent with the high electron mobilities reported in InSe-based FETs^12^,^13^ and provides a platform for 2D electronics and applications that require an additional in-plane carrier confinement. We have identified narrow quantum dot-like emissions with spin-splitting that cannot be resolved even at *B* = 30 T. Further studies are required to address this finding and the role of spin-orbit interaction that is expected to be weak in InSe^31^. Overall, InSe provides an interesting class of 2D systems that expands significantly the range of heterostructures and devices for electronic and photonic technologies.

## Methods

### Experimental techniques

The *γ*-polytype InSe crystals were grown using the Bridgman method from a polycrystalline melt of In_1.03_Se_0.97_. The crystal structure was probed by X-ray diffraction using a DRON-3 X-ray diffractometer in a monochromatic Cu-Kα radiation of wavelength *λ* = 1.5418 Å. The InSe nanosheets were prepared from the as-grown crystals by mechanical exfoliation. We used a two-step approach in which the flakes were first thinned down with the aid of F07 backgrinding tape from Microworld and then transferred onto a Si/SiO_2_ substrate by means of polydimethylosiloxane-based DGL-X4 elastomeric films from Gel-Pak. Images of the InSe flakes’ topography were acquired using a NSV-VEECO-D3100 atomic force microscope (AFM) operated in tapping mode under ambient conditions.

The experimental set-up for the μPL studies at *B* = 0 T comprised an Ar CW laser (*λ* = 514.5 nm), a He-Ne laser (*λ* = 633 nm) or a frequency doubled Nd:YVO_4_ laser (*λ* = 532 nm), an x-y-z motorized stage and an optical confocal microscope system equipped with a 0.5 meter long monochromator with 150, 300 and 1200 g/mm gratings. For experiments at *T* = 5 K, the sample was placed on the cold finger of a continuous gas flow cryostat mounted on an x-y-z motorized stage. The laser beam was focused to a diameter *d *~ 1 μm using 50× or 100× objectives. PL experiments were performed at low excitation power (*P* < 0.1 mW) to avoid excessive heating. The signal was detected by a charge-coupled device (CCD) camera.

The magneto-optical studies in the Faraday configuration were performed in a resistive magnet generating fields, *B*, up to 30 T. The sample was mounted on an x–y–z piezo-stage for precise (sub-micron) positioning. Optical fibres were used to transfer excitation light from an Ar CW laser (*λ* = 514.5 nm) and to collect the PL signal. The μPL set-up was placed in a probe, filled with He exchange gas and cooled to 4.2 K. The laser beam was focused to a diameter *d *~ 1 μm by a high numerical aperture lens. The detection of the signal was made using a 0.5 m monochromator with 300 g/mm grating and CCD camera. Due to the use of optical fibers in the system, the magnetic-field-induced rotation of the linear polarization angle (Faraday effect) introduces a modulation of the PL intensity.

### DFT calculations and Landau levels

In the *ab initio* DFT calculations of the band structure, we used the Perdew-Burke-Ernzerhof generalized gradient approximation exchange correlation functional for *L* = 1 layer^7^ and the optB88 vdW density functional for *L* > 1 layer. The first principle calculations were performed using the VASP code^32^ in a plane-wave basis. The cutoff energy for the basis set is 600 eV and we sampled the Brillouin zone with a regular Monkhorst-Pack grid using 12 grid points along each reciprocal lattice vector. To account for the interaction between the layers, we relies on the vdW density functional method of Klimeš *et al*. in the optB88 parametrization^33^,^34^. Single layers and bulk structures were fully relaxed until all forces fell below 5 meV/Å. Multilayer geometries were constructed from the bulk structure by neglecting surface relaxation effects, which are small: for *L* = 2 layers, the in-plane lattice parameter and inter-layer distance change by 0.07% and 0.06%, respectively. A comparison between measured and calculated band gap energies, *E*_*g*_, at *T* = 300 K indicates that DFT underestimates *E*_*g*_ by *δE* = 0.98 eV for *L* > 20 layers.

To calculate the LL spectrum, we fitted analytical expressions to the CB and VB energy dispersions as derived from DFT. The VB in the vicinity of Γ was approximated by an eighth-order polynomial function, 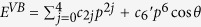, where *p* and *θ* are the polar coordinates of the quasi-momentum vector measured from Γ, and 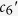, *c*_2*j*_ are constant parameters that depend on *L*. In this expression, all terms are isotropic around the zone center with the exception of the 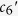 term, which is a small perturbation and accounts for the modulation of the “Mexican hat” dispersion according to a 6-fold rotational symmetry. The CB is instead isotropic and was described by 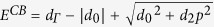, where *d*_*Γ*_, *d*_0_ and *d*_2_ depend on *L*. The Hamiltonian of the system in a magnetic field was then constructed introducing the vector potential using the Peierls substitution, *i.e*. the momentum operator 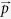 was replaced by 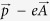, where 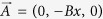 is the vector potential. The LL spectrum was obtained by solving the Schrödinger equation using a linear combination of LL harmonics, *ϕ*_*n*_(*x, y*) = *A*_*n*_*H*_*n*_(*x/λ*_*B*_ − *p*_*y*_*λ*_*B*_)exp[−(*x/λ*_*B*_ − *p*_*y*_*λ*_*B*_)^2^/2 + *ip*_*y*_*y*], where 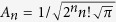, *H*_*n*_ are Hermite polynomials of order *n*, and 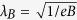.

### Data Availability

All relevant data are available from the University of Nottingham Data Repository, under the doi: https://doi.org/10.17639/nott.69.

## Additional Information

**How to cite this article**: Mudd, G. W. *et al*. The direct-to-indirect band gap crossover in two-dimensional van der Waals Indium Selenide crystals. *Sci. Rep.* **6**, 39619; doi: 10.1038/srep39619 (2016).

**Publisher's note:** Springer Nature remains neutral with regard to jurisdictional claims in published maps and institutional affiliations.

## Figures and Tables

**Table 1:** Calculated values of the in-plane electron (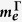), hole (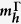 and 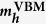) and exciton cyclotron masses (*μ*_⊥_^Γ^ and *μ*_⊥_^VBM^) for *L* = 1, 2, 5 and 10 InSe layers.

L number of layers	1	2	5	10	Bulk mass in the a-b plane
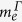 /m_e_	0.18	0.15	0.13	0.12	0.138^20^
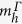 /m_e_	−0.70	−0.61	−0.68	−1.03	0.730^19^
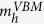 /m_e_	2.1	1.05	0.80	1.02	
μ_⊥_^Γ^/m_e_	0.14	0.12	0.11	0.11	0.14^24^
μ_⊥_^VBM^/m_e_	0.17	0.13	0.11	0.11	

The values for bulk γ-InSe are from the literature.
